# Genetic analysis of flag leaf size and candidate genes determination of a major QTL for flag leaf width in rice

**DOI:** 10.1186/s12284-014-0039-9

**Published:** 2015-01-17

**Authors:** Bin Zhang, Weijun Ye, Deyong Ren, Peng Tian, Youlin Peng, Yang Gao, Banpu Ruan, Li Wang, Guangheng Zhang, Longbiao Guo, Qian Qian, Zhenyu Gao

**Affiliations:** State Key Laboratory of Rice Biology, China National Rice Research Institute, Chinese Academy of Agricultural Sciences, Tiyuchang Road 359, Hangzhou, 310006 China

**Keywords:** Flag leaf size, Flag leaf width, QTL, Candidate genes, Rice

## Abstract

**Background:**

Flag leaf is the most essential organ for photosynthesis in rice and its size plays an important role in rice breeding for ideal plant-type. Flag leaf size affect photosynthesis to a certain extent, thereby influencing rice production. Several genes controlling leaf size and shape have been identified with mutants. Although a number of quantitative trait loci (QTLs) for leaf size and shape have been detected on 12 chromosomes with different populations of rice, few of them were cloned.

**Results:**

The pair-wise correlation analysis was conducted on length, width and length-width ratio of the flag leaf, and yield per plant in the core recombinant inbred lines of Liang-You-Pei-Jiu (LYP9) developed in Hainan and Hangzhou. There were significant correlations among the three flag leaf size and shape traits. Interestingly, a positive correlation was found between flag leaf width and yield per plant. Based on the high-resolution linkage map we constructed before, 43 QTLs were detected for three flag leaf size and shape traits and yield per plant, among which 31 QTLs were unreported so far. Seven QTLs were identified common in two environments. And *qFLW7.2*, a new major QTL for flag leaf width, was fine mapped within 27.1 kb region on chromosome 7. Both *qFLW7.2* and *qPY7* were located in the interval of 45.30 ~ 53.34 cM on chromosome 7, which coincided with the relationship between yield per plant (PY) and flag leaf width (FLW).

**Conclusion:**

*qFLW7.2*, which explained 14% of the phenotypic variation, increased flag leaf width with 93–11 allele. Two candidate genes were selected based on sequence variation and expression difference between two parents, which facilitated further QTL cloning and molecular breeding in super rice.

## Background

Rice is not only one of the most important food crops in China, but a staple food for more than half the world's population (Delseny et al. 2001). With increasing population, high yield has become one of targets in rice breeding. Photosynthesis is the primary source of grain yield in rice (Chen et al. 1995). The top three leaves of rice, particularly the flag leaf, are the main source of carbohydrates production (Abrol et al. 1993; Foyer, 1987). At least 50% of photosynthetic products for grain are provided by flag leaf, the most important organ for photosynthesis (Li et al. 1998). Some traits, such as size and shape of flag leaf, affect photosynthesis to a certain extent, thereby influencing production (Yue et al. 2006). Therefore, flag leaf shape is an index for ideal plant-type in rice breeding (Yang and Yang 1998; Yuan, 1997; Zhou et al. 1995).

Besides several genes controlling leaf size and shape cloned with mutants (Fujino et al. 2008; Qi et al. 2008; Zhang et al. 2009; Hu et al. 2010; Xiang et al. 2012), some QTLs for the traits of flag leaf size and rice yield have also been mapped with diverse populations, such as F_2_, doubled haploid (DH) and recombinant inbred lines (RILs) (Wang et al. 2004; Peng et al. 2007; Wang et al. 2009; Jiang et al. 2010a). Yan and Wang (1990) studied 11 flag leaf traits in *indica-japonica* hybrids, and argued that flag leaf length (FLL), FLW and flag leaf area (FLA) were controlled by two pairs of genes with at least more than 60% heritability. In recent years, with the rapid development of molecular markers and the increase in resolution of the linkage map, numbers of QTLs for flag leaf size and shape have been reported in rice. Li et al. (2000) detected 13 QTLs for FLL, FLW, FLA and length-width ratio (LWR), explained 8.7% ~ 18.5% of phenotypic variation, with DH population from a cross of Zhaiye Qing 8 and Jingxi 17. Using a DH population and a genetic map with 175 SSR markers under multi environments, Cao et al. (2007) detected 15 QTLs affected FLL, whose genetic intervals were 2 ~ 18 cM. Xiao et al. (2007) also identified 8 QTLs for the traits of FLL, FLW and FLA in the backcross recombinant inbred lines (BILs) derived from a cross between Koshihikari and Kasalath. However, most studies focused on the size and shape of the flag leaf and few involved in their relationship with yield. And so far, no QTL for flag leaf size has been isolated yet.

In the study, the relationship between flag leaf size and PY were analyzed. QTLs for three flag leaf traits and yield per plant (PY) were mapped based on a high-density linkage map by resequencing the parents of LYP9 and 132 core RILs (Gao et al., 2013). A novel major QTL for flag leaf width was fine mapped and 2 candidate genes were selected, by which providing a basis for further cloning of the QTL and improvement of ideal plant-type in hybrid rice.

## Results

### Phenotypic variation of the parents and RILs

**Figure 1:**
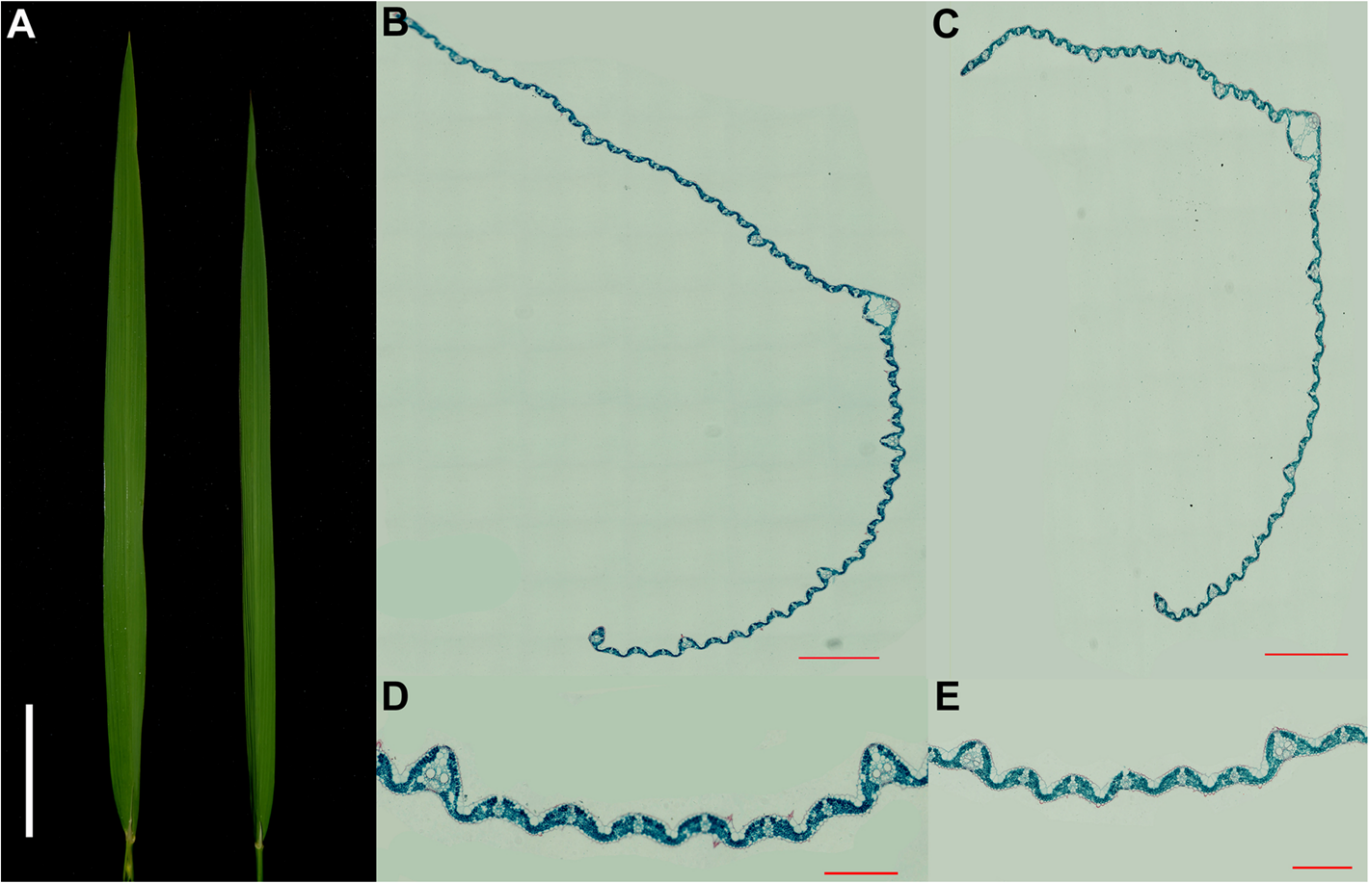
**Comparison of leaf morphology and transverse sections of flag leaf at heading stage between two parents. A**. Flag leaf of 93–11 (left) and PA64s (right). Bar = 5 cm. **B**, **D**. Paraffin section of flag leaf of 93–11. **C**, **E**. Paraffin section of flag leaf of PA64s. **B**, **C**. bar = 800 μm. **D**, **E**. bar = 200 μm.

**Figure 2:**
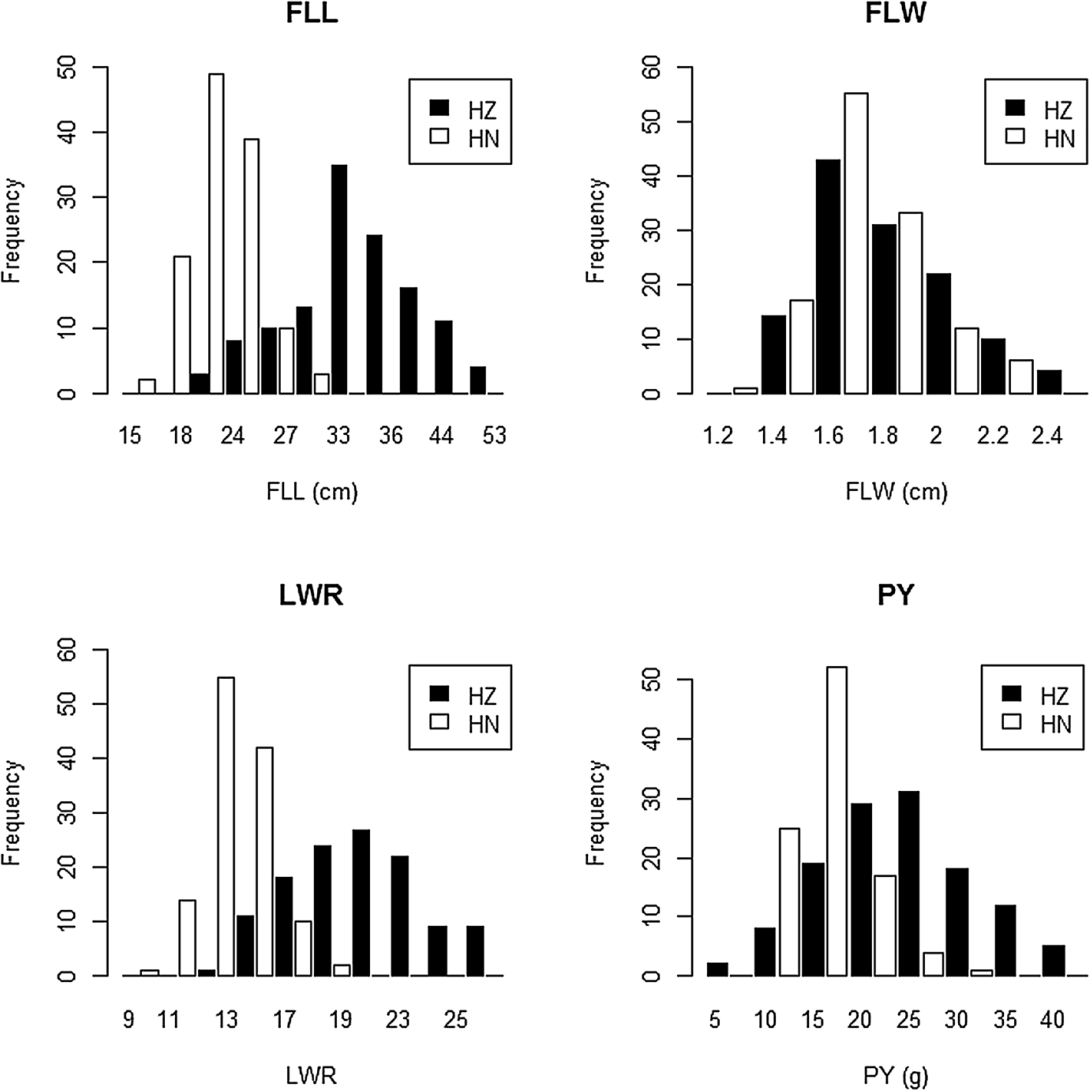
**Distribution of three flag leaf traits and plant yield in the RIL population.** HZ represents Hangzhou and HN represents Hainan.

The phenotypic differences between 93–11 and PA64s are displayed and summarized in Figure 1A and Table 1. The *t*-test revealed that the differences between two parents were extremely significant concerning FLW and PY in Hangzhou, as well as for FLL, FLW and PY in Hainan. While for FLL and LWR in Hangzhou, LWR in Hainan, the differences were significant between the parents. Microscopic observation with flag leaves indicated difference in the number of small veins on average between the parents, although little difference in the number of large veins (Figure 1B, C, D, E and Table 2). Continuous distributions were observed in the RIL population for all four traits and the average value of each trait was close to its mid-parent value (Figure 2), indicating all of the four traits were quantitative traits controlled by multi-genes and satisfied the demands for QTL analysis.

**Table 1:** **Variations of phenotypes between parents in Hainan and Hangzhou**

**Variety**	**FLL (cm)**	**FLW (cm)**	**LWR**	**PY (g)**
93-11-Hainan	25.23 ± 3.20**	1.95 ± 0.10**	12.94 ± 2.02*	19.20 ± 0.19**
PA64s-Hainan	20.46 ± 2.90	1.33 ± 0.10	15.38 ± 1.81	4.27 ± 0.24
93-11-Hangzhou	28.67 ± 3.80*	2.13 ± 0.10**	13.44 ± 2.56*	29.61 ± 0.18**
PA64s-Hangzhou	24.33 ± 3.70	1.47 ± 0.10	16.59 ± 2.45	0.00 ± 0.00

Mean ± SD (n = 6).

*and **indicate the least significant difference at 0.05 and 0.01 probability level compared with PA64s in Hangzhou or Hainan, respectively.

**Table 2:** **Numbers of large and small veins in flag leaf**

**Variety**	**Number of large veins per leaf**	**Number of small veins per leaf**
93-11	7.20 ± 0.84*	40.20 ± 1.92**
PA64s	6.00 ± 0.71	31.80 ± 1.48

Mean ± SD (n = 5). *and **indicate the least significant difference at 0.05 and 0.01 probability level compared with PA64s, respectively.

### Correlation analysis of the four traits

The correlations among the four characteristics were shown in Table 3. The results showed that significant positive correlations were detected between PY and FLW in both Hainan and Hangzhou. Meanwhile, FLL was positively correlated in extreme significance with FLW and LWR. Reasonably, negative correlations were identified between FLW and LWR in both Hainan and Hangzhou.

**Table 3:** **Correlation coefficients between three flag leaf traits and yield per plant**

**Traits in Hainan**	**FLL**	**FLW**	**LWR**
FLW	0.473**		
LWR	0.630**	−0.377**	
PY	0.160	0.210*	−0.090
**Traits in Hangzhou**	**FLL**	**FLW**	**LWR**
FLW	0.368**		
LWR	0.678**	−0.412**	
PY	0.070	0.222*	−0.107

*and **indicate the 5% and 1% significant level, respectively.

### QTL detection for flag leaf size and yield per plant

**Figure 3:**
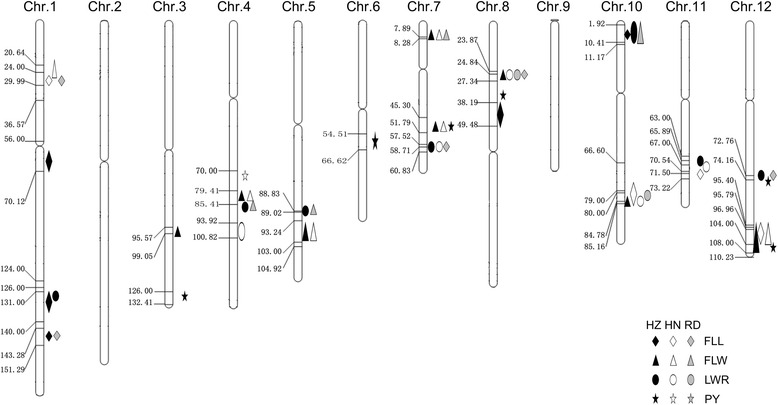
**Locations of QTLs on SNP map. Number indicates genetic distance (cM) along each chromosome.** HZ represents Hangzhou, HN represents Hainan and RD represents reported QTL.

A total of 43 QTLs were detected for the traits of FLL, FLW, LWR and PY in both Hainan and Hangzhou, distributing on 10 chromosomes except for chromosome 2 and chromosome 9 (Table 4; Figure 3). Nine QTLs for FLL were identified, including 4 QTLs in Hainan and 5 QTLs in Hangzhou, each explained 4% ~ 11% of phenotypic variation. Fourteen QTLs for FLW were detected and each QTL explained 4% ~ 24% of phenotypic variation. In Hangzhou, the 93–11 alleles increased FLW at *qFLW8* and *qFLW7.2*, explained 24% and 17% of the phenotypic variation, respectively. In Hainan, the 93–11 alleles also increased FLW at *qFLW1* and *qFLW7.2* which explained 15% and 14% of the phenotypic variation, respectively. For the trait of LWR, 12 QTLs were detected including 5 QTLs in Hainan and 7 QTLs in Hangzhou. Eight QTLs were identified for PY in both Hainan and Hangzhou. In Hangzhou, the 93–11 allele increased PY at *qPY7* which can explain 10% of the phenotypic variation and located within 49.20 ~ 53.34 cM on chromosome 7.

**Table 4:** **QTLs for four traits detected in RIL population in Hainan and Hangzhou**

**Traits**	**QTL**	**Site**	**Chr.**	**LOD**	**Marker interval**	**Genetic distance (cM)**	**A** ^**a**^	**PVE (%)** ^**b**^	**Reported QTL**
	*qFLL1*	Hainan	1	2.84	C1.loc24 ~ C1_9329218	24 ~ 36.57	0.59	4	*qFll1*(Tong et al*.* 2007)
	*qFLL10*	Hainan	10	2.90	C10_14487894 ~ C10.loc82	66.60 ~ 80.78	0.91	10	
	*qFLL11*	Hainan	11	3.82	C11.loc67 ~ C11_24850981	66.86 ~ 73.22	−0.96	11	
	*qFLL12*	Hainan	12	2.87	C12_24654159 ~ C12.loc108	95.40 ~ 108.13	−0.68	6	
FLL	*qFLL1.1*	Hangzhou	1	2.74	C1.loc56 ~ C1_19351415	56.65 ~ 70.12	1.72	8	
	*qFLL1.2*	Hangzhou	1	3.92	C1.loc124 ~ C1.loc140	124.21 ~ 133.43	−1.87	10	
	*qFLL1.3*	Hangzhou	1	3.32	C1_39017544 ~ C1_39489223	143.28 ~ 151.29	−1.95	5	*fll1*(Yan et al. 1999)
	*qFLL8*	Hangzhou	8	3.70	C8_9083764 ~ C8_10724396	38.19 ~ 49.48	1.75	8	
	*qFLL10*	Hangzhou	10	2.69	C10_706046 ~ C10_1469028	1.92 ~ 10.41	1.59	6	*qFLW10*(Li et al. 2010)
	*qFLW1*	Hainan	1	5.28	C1_6803535 ~ C1_7849762	20.64 ~ 29.99	0.08	15	*qFlr1*(Tong et al*.* 2007)
	*qFLW4*	Hainan	4	5.29	C4_23377395 ~ C4_23560797	84.83 ~ 85.41	0.05	6	
	*qFLW5*	Hainan	5	3.88	C5_24207944 ~ C5.loc103	93.24 ~ 103.16	−0.04	3	
	*qFLW7.1*	Hainan	7	4.20	C7_4865508 ~ C7_4925247	7.89 ~ 8.28	0.04	3	
	*qFLW7.2*	Hainan	7	4.25	C7_22333409 ~ C7_25017224	45.68 ~ 51.79	0.08	14	
	*qFLW12*	Hainan	12	2.99	C12_24691752 ~ C12.loc104	95.79 ~ 104.66	−0.07	10	
FLW	*qFLW3*	Hangzhou	3	3.49	C3_29306491 ~ C3_29977886	95.57 ~ 99.05	−0.09	12	
	*qFLW4*	Hangzhou	4	3.26	C4_22748438 ~ C4_23560797	79.41 ~ 85.41	0.06	6	*qFLW4.1*(Xu et al*.* 2011)
	*qFLW5*	Hangzhou	5	3.85	C5_24207944 ~ C5_26190467	93.24 ~ 104.92	−0.08	10	
	*qFLW7.1*	Hangzhou	7	4.80	C7_4865508 ~ C7_4925247	7.89 ~ 8.28	0.04	3	*qFLW7-1*(Li et al*.* 2010)
	*qFLW7.2*	Hangzhou	7	5.13	C7_22297400 ~ C7_25017224	45.30 ~ 51.79	0.11	17	
	*qFLW8*	Hangzhou	8	7.45	C8_4613627 ~ C8_5260282	24.84 ~ 27.34	0.13	24	
	*qFLW10*	Hangzhou	10	4.66	C10_18696371 ~ C10_18804231	84.78 ~ 85.16	0.04	2	*qFLWR10*(Zhou et al*.* 2012)
	*qFLW12*	Hangzhou	12	3.31	C12_25189929 ~ C12_26963973	96.96 ~ 110.23	−0.10	12	
	*qLWR4*	Hainan	4	6.13	C4_26804875 ~ C4_25808877	93.92 ~ 100.82	−0.62	13	
	*qLWR7*	Hainan	7	3.26	C7_27035206 ~ C7_27020954	57.52 ~ 58.71	0.17	1	
	*qLWR8*	Hainan	8	2.72	C8.loc28 ~ C8_4544399	23.87 ~ 28.30	−0.13	1	*qFlr8*(Tong et al*.* 2007)
	*qLWR10*	Hainan	10	3.67	C10.loc80 ~ C10.loc79	79.25 ~ 80.02	0.47	7	
	*qLWR11*	Hainan	11	3.33	C11_24536879 ~ C11.loc63	63.79 ~ 71.50	−0.53	10	
	*qLWR1*	Hangzhou	1	4.83	C1.loc126 ~ C1.loc131	126.33 ~ 131.13	−1.11	9	
LWR	*qLWR4*	Hangzhou	4	3.93	C4_23257341 ~ C4_23560797	84.45 ~ 85.41	−1.19	10	
	*qLWR5*	Hangzhou	5	4.37	C5_22399125 ~ C5_22575173	88.83 ~ 89.02	0.72	4	*qFLW5.1*(Xu et al*.* 2011)
	*qLWR7*	Hangzhou	7	2.50	C7_27020954 ~ C7_27614442	57.52 ~ 60.83	−0.64	3	*qFLL7*(Li et al*.* 2010)
	*qLWR10*	Hangzhou	10	2.57	C10_706046 ~ C10_2088765	1.92 ~ 11.17	1.11	8	
	*qLWR11*	Hangzhou	11	3.01	C11_23743973 ~ C11_24330376	65.89 ~ 70.54	−0.56	2	
	*qLWR12*	Hangzhou	12	5.61	C12_21654866 ~ C12_21692352	72.76 ~ 74.16	−1.09	8	*qFL12*(Zhou et al*.* 2012)
	*qPY1*	Hainan	1	3.97	C1_27996574 ~ C1_28029950	105.67 ~ 106.26	0.45	2	
	*qPY4*	Hainan	4	6.30	C4.loc70 ~ C4.loc72	68.97 ~ 72.09	−0.85	8	
	*qPY3*	Hangzhou	3	2.58	C3.loc125 ~ C3_35974986	126.09 ~ 132.41	2.60	9	
PY	*qPY6*	Hangzhou	6	3.34	C6_23535296 ~ C6_27331925	54.51 ~ 66.62	3.33	11	Unnamed(Jiang et al*.* 2004)
	*qPY7*	Hangzhou	7	2.61	C7_22387620 ~ C7_25413216	49.20 ~ 53.34	2.95	10	
	*qPY8*	Hangzhou	8	2.92	C8_4060421 ~ C8_8591477	23.29 ~ 36.27	2.72	10	
	*qPY12.1*	Hangzhou	12	2.75	C12_21588194 ~ C12_23465426	72.18 ~ 88.24	2.70	8	
	*qPY12.2*	Hangzhou	12	2.75	C12.loc103 ~ C12.loc105	103.90 ~ 105.05	−1.03	2	

^a^Additive effects; The positive value indicates that alleles from 93–11 increase the effect.

^b^PVE is the percentage of phenotypic variation explained by the detected QTL.

Among all the 43 QTLs detected with RILs, 7 QTLs were commonly identified in both Hainan and Hangzhou, demonstrating their environmental independent. Five of them distributing on chromosome 4, 5, 7, 7 and 12 were responsible for FLW and the other two QTLs on chromosome 7 and 11 for LWR. There were 10 clusters involving at least two QTLs, among which three clusters on chromosome 7, 8 and 12 simultaneously responsible for FLW and PY, which coincided with significant correlations between the two traits.

### Fine mapping and candidate gene analysis of a major QTL *qFLW7.2*

**Figure 4:**
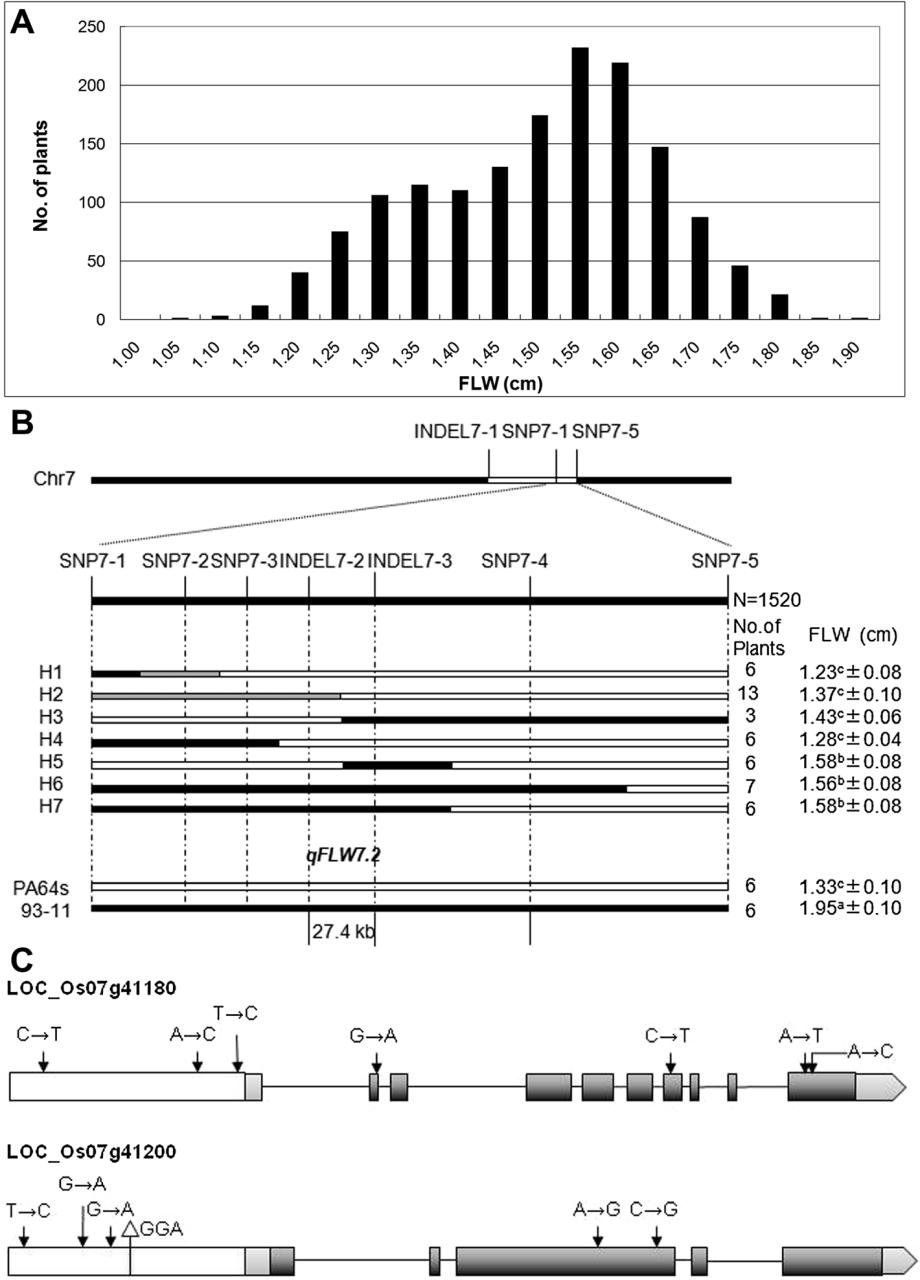
**Fine mapping of qFLW7.2 for FLW. A**. Distribution of FLW in the F_2_ population derived from RHL. **B**. *qFLW7.2* was narrowed down to a 27.4 kb interval defined by markers INDEL7-2 and INDEL 7–3. Values represent means ± SD. Gray represents heterotype. The superscript letters (a, b and c) indicate significant differences in the trait of the recombinants compared with two parents at a level of 0.01. **C**. Structure and mutated sites of two candidate genes. Grey boxes represent exons.

**Figure 5:**
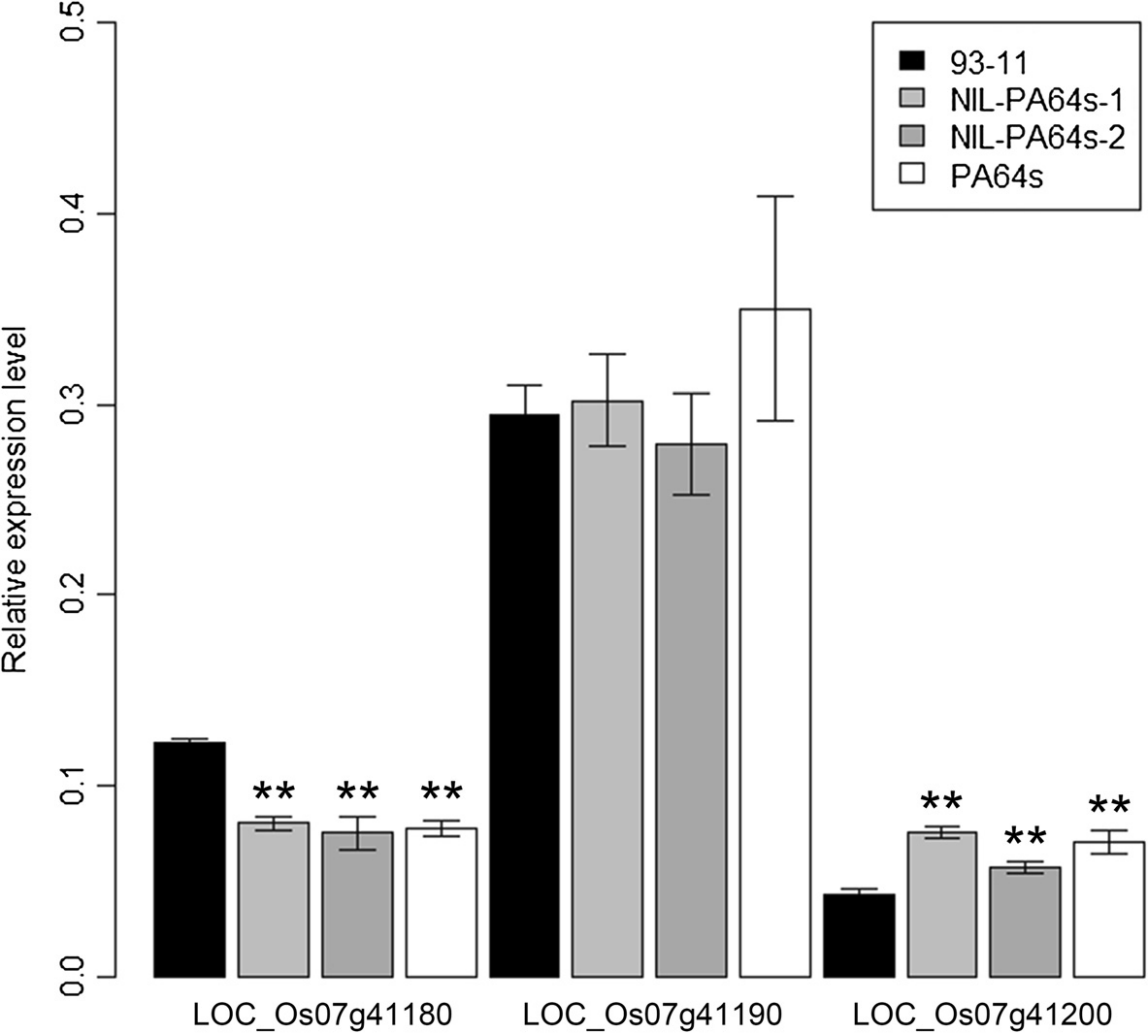
**Quantitative real-time RT-PCR analysis of predicted genes in flag leaves of two parents and two NILs at booting stage.** Values represent means ± SD of three independent assays. **indicates the 1% significant level.

Among 43 QTLs detected in RILs, 31 QTLs were unreported so far, including *qFLW7.2* identified in both Hainan and Hangzhou. For fine mapping of the new major QTL, residual heterozygous line (RHL) were selected from a large RIL population, carrying approximately 484 kb heterozygous segment on the long arm of chromosome 7. Then phenotypic character was measured in F_2_ population including 1520 individuals derived from the RHL. Three insertion-deletion (InDel) and five single nucleotide polymorphism (SNP) markers were developed by comparing the sequences of the parents. Combining the genotype and phenotype of individuals, the QTL was delimited between two InDel markers INDEL7-2 and INDEL7-3 in 27.1 kb interval (Figure 4B). The target region contains 3 predicted genes (*LOC_Os07g41180, LOC_Os07g41190* and *LOC_Os07g41200*) based on Rice Genome Annotation Project Website (http://rice.plantbiology.msu.edu/). Sequence variations of those genes between two parents were identified and expressions at RNA level were analyzed in leaves of the parents at booting stage (Figure 4C; Figure 5). Four SNPs causing amino acid change and 3 SNPs existed in exons and the promoter region, respectively in *LOC_Os07g41180* gene. And the gene *LOC_Os07g41200* had 2 nonsynonymous SNPs in one exon, 3 SNPs and an InDel in the promoter (Figure 4C). Both genes expressed at significantly different level in PA64s and two NILs (NIL-PA64s-1 and NIL-PA64s-2) compared with 93–11 (Figure 5). There were only 6 SNPs in the promoter of *LOC_Os07g41190* gene and no significantly different expression in PA64s and two NILs compared with 93–11. Therefore, *LOC_Os07g41180* and *LOC_Os07g41200* were selected candidates for *qFLW7.2*.

**Figure 6:**
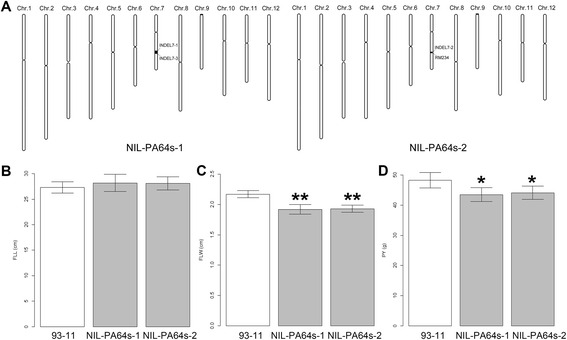
**Comparison in FLL, FLW and PY of NIL-PA64s-1, NIL-PA64s-2 and 93-11.** **A**. Schematic graph of chromosomes of NIL-PA64s-1 and NIL-PA64s-2. **B**, **C**, **D**. Comparison of FLL, FLW and PY between NIL-PA64s-1, NIL-PA64s-2 and 93–11. NIL-PA64s-1 and NIL-PA64s-2, carrying homozygous alleles of PA64s in the target QTL region (black box) between INDEL7-1 and INDEL7-3, INDEL7-2 and RM234, respectively, were developed from one CSSL with 93–11 background. Values represent means ± SD of three plants.

Comparison of flag leaf size between the two near isogenic lines (NILs) and 93–11 revealed FLW was wider in 93–11 than in NIL-PA64s-1 and NIL-PA64s-2, while little difference found between 93–11 and two NILs in FLL (Figure 6A, B, C). Meanwhile, significant difference was observed between 93–11 and two NILs in PY (Figure 6D). It indicated that the allele from PA64s affect flag leaf width and yield per plant at *qFLW7.2* between INDEL7-2 and INDEL7-3, approximately 27.1 kb physical distance (Figure 6A).

## Discussion

Leaf is the main organ for photosynthesis in rice. Several rice mutants for leaf size and shape have been identified and some corresponding genes have been cloned. Fujino et al. (2008) isolated a spontaneous mutant with narrow leaf, termed *narrow leaf 7* (*nal7*). The gene *Nal7*, encoding a flavin-containing mono-oxygenase, were fine mapped on chromosome 3 and cloned with F_2_ population. The *Nal1* gene located on chromosome 4, whose mutation affected lateral leaf growth and exhibited narrow leaf, encodes a plant specific protein of unknown biochemical function (Qi et al. 2008). And the *NRL1* gene was fine mapped on the chromosome 12 and coded for the cellulose synthase-like protein D4 (Hu et al. 2010). Currently, many QTLs have been fine mapped related to flag leaf size and shape. Jiang et al. (2010b) detected 3 QTLs for FLL on chromosome 3, 6, and 9 using a separated population involving 176 individuals from a cross of Shennong 265/LTH. And *qFLL9* was further mapped within a 198 kb interval on chromosome 9 by analyzing F_2_ population including 889 individuals derived from the RHL. Shen et al. (2012) fine mapped *qFLL6.2* within 62.1 kb on the short arm of chromosome 6 by a F_2_ population derived from the RHL. Wang et al. (2011) narrowed the location of *qFL1* for flag leaf length to a 31 kb region containing 4 predicted genes with BC_2_F_3_ and BC_3_F_2_. In our study, 43 QTLs were detected in two environments with the shortest genetic interval 0.19 cM in a high-density linkage map using the RIL population. Among 31 unreported QTL, a novel major QTL *qFLW7.2*, detected in both Hainan and Hangzhou was fine mapped in a 27.1 kb physical interval on chromosome 7 with the F_2_ population derived from a RHL. Two NILs containing the region from PA64s also showed narrower FLW compared with 93–11. Two candidate genes, *LOC_Os07g41180* and *LOC_Os07g41200*, encoding RNA-binding protein and unknown expressed protein respectively, were selected based on sequence variations and transcriptional expression and to be further testified by complementation test.

As the most important and efficient functional leaf at grain filling stage, flag leaf shape is one of the essential traits for ideal plant-type in super rice breeding (Chen et al. 2001). It played an important role in molecular genetics and marker assisted selection (MAS) of flag leaf size and shape related traits. Here, PY and FLW were found significantly and positively correlated, which suggested that appropriate increase in FLW may raise PY correspondingly. Both *qFLW7.2* and *qPY7* were located in the interval of 45.30 ~ 53.34 cM on chromosome 7, which coincided with the relationship between PY and FLW. It suggested that *qFLW7.2* may show pleictropism and play an important role in the formation of rice yield. Previous studies found that FLW was significantly correlated to panicle number and spikelet number per panicle (Zhou et al. 2012). Recent studies showed that *SPIKE*, a *nal1* allele, can increase 13 ~ 36% of the yield of the NIL derived from *indica* variety IR64, with leaf area significantly increased. Therefore, *SPIKE* was believed to induce the enhancement of source size and translocation capacity as well as sink size (Fujita et al. 2013). Therefore, it was reasonable that wider flag leaf may increase photosynthetic area, so that the source supply was enhanced, and thereby rice yield improved. Moreover, molecular markers adjacent to *qFLW7.2* can also be utilized effectively in controlling flag leaf width and high-yield breeding in rice.

## Conclusion

In this study, using high-density SNP linkage map, 43 QTLs were detected in Hangzhou and Hainan to control rice leaf morphology and yield per plant. Owing to the increased precision and sensitivity of detection, minimum QTL interval reached 0.19 cM and 31 QTLs were novel. With the F_2_ population derived from a RHL, *qFLW7.2*, a new major QTL for FLW, was fine mapped within 27.1 kb physical interval on chromosome 7. Two candidate genes were finally selected based on difference in genomic sequence and transcriptional expression. Because the significantly positive correlation between FLW and PY, together with common interval shared by QTLs for FLW and PY, appropriate increase in FLW may raise PY correspondingly during molecular breeding for ideal plant-type in rice.

## Methods

### Mapping population and genetic map

The core mapping population of 132 LYP9 RILs was derived by single-seed descend from a cross between an elite paternal inbred *Oryza sativa. indica* cv. 93–11 and the maternal inbred *Oryza sativa. javonica* cv. Peiai 64 s (PA64s), a photo-thermo-sensitive male sterile line. The population was developed in the experimental fields at China National Rice Research Institute in Hangzhou, Zhejiang Province and in Lingshui, Hainan Province, China. After 12 generations of self-fertilization, genomic DNA samples of the F_13_ RILs were isolated for genotyping. High-density map of genome-wide graphic genotypes was constructed using single nucleotide polymorphism SNP markers as described previously (Gao et al*.*, 2013). The RHL carrying approximately 484 kb heterozygous segment on the long arm of chromosome 7 was segregated from large high-generation RILs with 1520 individuals. A F_2_ population derived from the RHL was used for fine mapping. Two NILs carrying homozygous alleles of PA64s in the target QTL region between InDel markers INDEL7-1 and INDEL7-3, INDEL7-2 and a simple sequence repeat (SSR) marker RM234 (Tian et al., 2013), designated respectively NIL-PA64s-1 and NIL-PA64s-2, were also developed from one chromosome segment substitution line (CSSL) with 93–11 background (Table 5).

**Table 5:** **Primers for InDel markers and SNP markers developed**

**Primer**	**Forward (5’-3’)**	**Reverse (5’-3’)**	**Product length (bp)**	**Annealing temperature (°C)**
INDEL7-1	tcgataaaagttcagtttgacggc	actttttcatccgcgacgaatatc	68 (62)*	55
INDEL7-2	tgaagtggcatgatccatctacac	tgtactgcactgcagtggatgc	81 (75)*	55
INDEL7-3	tttttagattatttacttcacg	taatcaagaaggacttttgag	65 (69)*	50
SNP7-1	tcggattcaatgtgtcactctc	acatgctactagttattcctcgtaaac	111	58
SNP7-2	tgacgcattctcgatggagtc	tatcggggacttgttctcattc	80	58
SNP7-3	aggaataccagatgctgttgtcg	aactccccctccagtgtagcc	78	60
SNP7-4	tcaaagacatgacatcacgacac	cagagcacctataagtaacagtctaac	84	58
SNP7-5	tcattagcacatatttattgtagcacc	gaaaaaaccaattacacagattgc	106	60

*Number in brackets indicates the product length of PA64s.

### Field experiment and trait measurement

The 132 RILs and two parental lines were grown in Hangzhou in 2011 and Hainan in 2012. The F_2_ population derived from the RHL was grown in Hangzhou in 2013. NILs were cultivated in Hangzhou in 2014 following a randomized block design with three repeats. 25-day-old seedlings of each line were transplanted into a four-row plot with six plants per row and spacing of 15 cm × 25 cm. The field management followed normal agricultural practice.

Three flag leaf size and shape traits were investigated for four plants per line in the middle of rows 10 days after heading. The flag leaf length (FLL, cm) and flag leaf width (FLW, cm) were measured on three tillers. One derived trait, the length-width ratio (LWR) = FLL/FLW, was calculated. The trait yield per plant (PY, g) was also examined for the plants whose flag leaf size and shape had been investigated.

### Leaf sections and microscopic analysis

Flag leaves of two parents were collected at heading stage and fixed in Formalin-Aceto-Alcohol (FAA). The samples were dehydrated through a graded ethanol series, then embedded in Paraffin (Surgipath®) and polymerized at 60°C. Finally, the materials were sectioned and stained with 1% toluidine blue before examination under an ECLIPSE 50i microscope (Nikon) (Hu et al. 2010).

### QTL analysis

Phenotypic variations and correlations were analyzed by SAS 8.0 software. QTL analysis was performed with the R/qtl_1.26-14 (http://www.rqtl.org/) using Composite Interval Mapping (CIM). LOD threshold for each dataset was set based on a permutation test (1,000 permutation, P = 0.05). It was considered as a major effect QTL when its LOD score was larger than 2.5. PEV was estimated by ANOVA. QTLs were named according to McCouch et al*.* (1997).

### Development of InDel and SNP markers for fine mapping

Primers were designed around *qFLW7.2* on chromosome 7 on the basis of insertions/deletions (InDels) and SNPs identified between 93–11 and PA64s (Table 5). Genotypes of SNP markers were screened by high-resolution dissociation curve analysis system (LightScanner 96, Idaho Technology Inc.).

### RNA extraction and real time PCR analysis

Total RNA was isolated from flag leaf at booting stage with RNA extraction kit (Axygen). DNase treatment, cDNA synthesis, primer design and SYBR Green I real time PCR were carried out as described (Vandesompele et al. 2002) using a Rever Tra Ace® qPCR-RT kit (TOYOBA, Japan). Real time PCR amplification mixtures (10 μl) contained 50 ng template cDNA, 2 × SYBR Green PCR Master Mix (Applied Biosystems), and 200 nM forward and reverse primers. Reactions were run on an ABI PRISM_7900HT Sequence Detector (Applied Biosystems). The relative expression level of each transcript was obtained by comparing to the expression of the *OsActin1* gene. Primers for candidate genes and *OsActin1* are listed in Table 6.

**Table 6:** **Primers for real time PCR analysis**

**Gene**	**Primer**	**Forward (5’-3’)**	**Reverse (5’-3’)**	**Product length (bp)**
*LOC_Os07g41180*	RT-1	gcatccattgttgaggagaaacg	cacctctgttgtcttgctggaac	112
*LOC_Os07g41190*	RT-2	cctcaagatgaatgggaatgtgcgt	tacacttccttgtcctgagatccca	116
*LOC_Os07g41200*	RT-3	gagaatgccccaagtcccatctc	ctgttcgggttccagcactc	116
*OsActin1*	RT-4	ccattggtgctgagcgttt	cgcagcttccattcctatgaa	70
